# UHPLC-HRMS–based serum lipisdomics reveals novel biomarkers to assist in the discrimination between colorectal adenoma and cancer

**DOI:** 10.3389/fonc.2022.934145

**Published:** 2022-07-28

**Authors:** Hongwei Chen, Jiahao Zhang, Hailin Zhou, Yifan Zhu, Yunxiao Liang, Pingchuan Zhu, Qisong Zhang

**Affiliations:** ^1^ Medical College of Guangxi University, Guangxi University, Nanning, China; ^2^ Department of Gastroenterology, People’s Hospital of Guangxi Zhuang Autonomous Region, Nanning, China; ^3^ State Key Laboratory for Conservation and Utilization of Subtropical Agro-Bioresources, Guangxi University, Nanning, China

**Keywords:** colorectal adenoma, colorectal cancer, biomarkers, lipidomics, UHPLC-HRMS

## Abstract

The development of a colorectal adenoma (CA) into carcinoma (CRC) is a long and stealthy process. There remains a lack of reliable biomarkers to distinguish CA from CRC. To effectively explore underlying molecular mechanisms and identify novel lipid biomarkers promising for early diagnosis of CRC, an ultrahigh-performance liquid chromatography tandem high-resolution mass spectrometry (UHPLC-HRMS) method was employed to comprehensively measure lipid species in human serum samples of patients with CA and CRC. Results showed significant differences in serum lipid profiles between CA and CRC groups, and 85 differential lipid species (*P* < 0.05 and fold change > 1.50 or < 0.67) were discovered. These significantly altered lipid species were mainly involved in fatty acid (FA), phosphatidylcholine (PC), and triacylglycerol (TAG) metabolism with the constituent ratio > 63.50%. After performance evaluation by the receiver operating characteristic (ROC) curve analysis, seven lipid species were ultimately proposed as potential biomarkers with the area under the curve (AUC) > 0.800. Of particular value, a lipid panel containing docosanamide, SM d36:0, PC 36:1e, and triheptanoin was selected as a composite candidate biomarker with excellent performance (AUC = 0.971), and the highest selected frequency to distinguish patients with CA from patients with CRC based on the support vector machine (SVM) classification model. To our knowledge, this study was the first to undertake a lipidomics profile using serum intended to identify screening lipid biomarkers to discriminate between CA and CRC. The lipid panel could potentially serve as a composite biomarker aiding the early diagnosis of CRC. Metabolic dysregulation of FAs, PCs, and TAGs seems likely involved in malignant transformation of CA, which hopefully will provide new clues to understand its underlying mechanism.

## Introduction

Colorectal cancer (CRC) is one of the most common malignant tumors with a continued high mortality worldwide (1). It was estimated that there were more than 1.9 million new colorectal cases (including anus) and 935,000 deaths worldwide in 2020, ranking third in incidence (10.0% of the total cancer cases) and second in mortality (9.4% of total cancer deaths) (2). The earliest stage of CRC has no specific symptoms, which can delay diagnosis (3), resulting in less effective treatment, and increased risk of surgical or chemotherapy side effects, particularly in the mid to late disease stages, and consequently higher mortality. Studies have documented that the inception and development of CRC typically follow an “adenoma to carcinoma” sequence, which generally require 10–15 years to complete. Therefore, the gross and even microscopic characteristics of colorectal adenomas (CAs) rarely show changes indicative of transformation into CRC (4, 5). Early screening and resection of CA is crucial to prevent cancer and reduce the mortality rate of CRC by as much as 50% (6). Currently, colonoscopy has been widely accepted as the “gold standard” in clinical practice as a screening method for CA and CRC and has been effective in reducing mortality (7). Nevertheless, colonoscopy does have a low but significant risk of complications, such as abdominal pain, bleeding, and perforation (8). Moreover, colonoscopy is also limited by its invasiveness, modest patient compliance, and high technical and cost requirements (9). Comparatively, detection based on minimally invasive or non-invasive diagnostic biomarkers offers a simplified method that enhances patient compliance (10). At present, there is still a lack of minimally invasive biomarkers to discriminate the CA and CRC for the early screening of CRC, Hence, it is urgent to discover novel biomarkers, aiding the discrimination between CA and CRC.

Mass spectrometry–based high-throughput analytical technology has been widely used recently to identify new biomarkers of disease from numerous biological molecules (11, 12). Following investigations of genomics, transcriptomics, and proteomics, metabolomics as one of the typical representatives of high-throughput mass spectrometry technique has attracted great attention for its application in screening, detection, and diagnosis of tumors (13). By using biofluids (especially serum or plasma), the study techniques for metabolome profiling assure a systematic screening or fingerprinting of metabolites with molecular weight < 1,000 Da related to the metabolic signature and pathway alterations in various stages of disease to discover the putative biomarkers and mechanisms (14). Among the detected metabolites, lipid species have recently become the focus of many metabolomic studies, owing to relatively mature technology and well-developed databases, leading to the development of a whole discipline, lipidomics (15). As a key branch and advanced methodology of metabolomics, lipidomics systematically and comprehensively investigates the changes in lipid profiling and related lipid metabolic pathways within organisms under different physiological or pathological states (16).

The pathogenesis of CRC is accompanied by dysregulation of various endogenous metabolic pathways. Effective monitoring of changes in endogenous metabolites can quickly and accurately reflect the physiological or pathological state of the body. Lipid species are widely distributed and important endogenous substances *in vivo* that have been demonstrated to be involved in the occurrence and development of various tumors (17, 18). Recent studies have reported that perturbation of lipid metabolism is closely related to the progression of CRC (19, 20). The lipid metabolic pathways in CRC cells are affected, including fatty acid synthesis, desaturation, elongation, and mitochondrial oxidation (21). A plasma lipidomic study revealed that glycerolipid and glycerophospholipid metabolism as well as sphingolipid metabolism were demonstrably perturbed in patients with CRC (22). Tissue lipidomic research found that lysophosphatidylcholines (LPCs) and phosphatidylcholines (PCs) were the most strongly related biomarkers of CRC establishment (23). However, no lipidomic studies have been conducted that have reported the lipid biomarkers that can discriminate between CRC and CA.

The intent of this study was to undertake an untargeted lipidomic study utilizing serum samples from patients with CRC and CA, based on ultrahigh-performance liquid chromatography tandem high-resolution mass spectrometry (UHPLC-HRMS) to produce global lipid profiling between the two groups of patients. Through combining multivariate and univariate statistical methods, the inevitable differences in the serum lipid species should permit discrimination between the lipid profiles of the CA and CRC groups, eventually to identify lipid biomarkers to aid in the early detection and prevention of CRC. Ultimately, the mechanism of lipid metabolic pathways contributing to malignant transformation of CA would be better understood.

## Materials and methods

### Chemicals and reagents

HPLC grade methanol, dichloromethane, isopropanol, and acetonitrile were purchased from Merck & Co. (Billerica, MA, USA). Ultrapure water was prepared by a Millipore Milli-Q system (Billerica, MA, USA).

### Study cohort and sample collection

Serum samples of 50 patients with CRC and 50 patients with CA were collected from the People’s Hospital of Guangxi Zhuang Autonomous Region. All subjects were recruited in the present study with the same sample collection protocol. The protocol of this study was approved by the Ethics Committee of Guangxi Zhuang Autonomous Region People’s Hospital (No. KY-DZX-202008), including the collection of detailed information about serum samples and subjects as well as the written consent of all subjects in the study. The main clinical and demographic characteristics of enrolled subjects were shown in **Table 1**. In addition, possible differences related to sex and age were assessed by the Chi-square test and Student’s *t*-test, which showed no statistically significant differences in these two physiological measures between the two groups. Inclusion criteria for patients with CRC were as follows. (1) Patients were proven to suffer from CRC by pathology. (2) Patients were not attacked with any metabolic diseases, such as diabetes, kidney, liver diseases, or other cancers. (3) Patients had not received chemotherapy or radiotherapy prior to this study. (4) Patients must have the accurate and detailed clinical information. The inclusion criteria of CA patients were as follows. (1) Patients were diagnosed as CA by colonoscopy and pathology. (2) Patients were not attacked with severe metabolic or hematologic diseases or malignancy. All whole-blood samples were taken after an 8-h fast, left to stand at room temperature for 25 min, and serum was then collected following centrifugation at 5,000 rpm/min for 10 min at 4°C. The serum samples were immediately stored at −80°C condition until analysis.

**Table 1:** Basic clinical information of study subjects.

Group	Gender (Male/Female)	Age (year)	Position	Vienna Classification	Differentiation	Tumor Stage
**CA** (N = 50)	18/32	56 ± 12	Rectum (23) Colon (27)	High (26) Low (24)		
**CRC** (N = 50)	23/27	61 ± 9	Rectum (11) Colon (29)		High (0) Middle (37) Low (6) Unknown (7)	0 stage (1) I stage (11) II stage (21) III stage (15) Unknown (2)
***P*-value** ^a^	0.416	0.213				

CA, colorectal adenoma; CRC, colorectal cancer. ^a^ Chi-square test and Student’s t-test.

### Sample preparation

For serum sample preparation, the detail method was referred to our previous study (12). Precooling dichloromethane-methanol (3:1, v/v) solution (500 μl) was added to 50 μl of serum. After being vortexed for 5 min and placed in an ice bath for 10 min, the solution was centrifuged at 13,000 rpm/min at 4°C for 10 min. Lower dichloromethane solution (300 μl) was dried in vacuum at room temperature. The dried samples were redissolved with 600 μl of acetonitrile-isopropanol (1:1 v/v) solution and then vortexed for 2 min and ultrasonicated in an ice bath for 5 min. Following vortexed for 1 min, the vials were centrifuged at 13,000 rpm/min at 4°C for 15 min, and the supernatants were collected for lipidomics analysis. Quality control (QC) samples were prepared by the same pretreatment method by mixing 5 μl of each sample. Before formal sample analysis, the repeatability and stability of the analytical system and method were verified by balancing the analytical system with at least six blank samples and six QC samples.

### UHPLC-HRMS–based lipidomic analysis

Serum lipidomic analysis was performed using Dionex Ultimate 3000 liquid chromatography system (Sunnyvale, CA, USA) (SN: 7254012) coupled to Thermo Fisher Q Exactive Orbitrap mass spectrometry system (Waltham, MA, USA) (SN: SN02386L), operated in positive and negative ionization modes, respectively. Liquid chromatography system equipped with a Waters Acquity UHPLC HSS T3 column (1.8 μm, 2.1 × 100 mm; Milford, MA, USA) was applied for the separation analysis of prepared serum samples with the column temperature of 50°C. The mobile phase was represented by a gradient of eluent A (water: acetonitrile = 4:6, v/v, containing 0.1% formic acid and 10 mM ammonium formate) and eluent B (isopropanol: acetonitrile = 9:1, v/v, containing 0.1% formic acid and 10 mM ammonium formate) with flow rate of 0.3 ml/min. The gradient elution conditions were set as follows: 0.0–4.0 min, 30% to 60% B; 4.0–9.0 min, 60% to 100% B; 9.0–15.0 min,100% B; 15.0–18.0 min,100% B to 30% B. The MS spectrometric parameters were as follows: spray voltage, 3.5 kV; sheath gas flow rate, 50 psi; auxiliary gas flow rate, 13 arb; capillary temperature, 320°C; auxiliary gas heater temperature, 420°C; scan modes, full MS (resolution 70,000) and ddMS2 (resolution 17,500 with stepped collision energy (10, 20, and 40 eV); and scan range, *m/z* 100–1200. All data were acquired by using the Thermo Scientific Xcalibur 3.1 software (Waltham, MA, USA).

### Data processing and statistical analyses

The data processing procedures of serum lipidomics were based on our previous study (12). For univariate statistical analysis, raw data files were imported into the Thermo Scientific Compound Discoverer™ 3.1 software for data analysis. Lipidomic three-dimensional data were extracted and the data normalization was conducted by using QC samples to effectively uncover differential lipid species. The differential analysis between groups was analyzed with Mann–Whitney *U*-test or Student’ *t*-test. The lipid feature with *P* < 0.05 was indicated significant differences between the two groups. Potential lipid species were identified depending on Thermo mzVault and LipidBlast database. The main parameters of data processing were as follows: minimal peak intensity, 500,000; mass error, 10 ppm; RT tolerance, 0.2 min; intensity tolerance, 20%; and S/N, 3.

For multivariate statistical analysis, principal component analysis (PCA) and orthogonal partial least squares discriminant analysis (OPLS-DA) were performed with the software SIMCA-*P* 14.1 (Umetrics, UMEA, Sweden). A 200 times permutation test was carried out to avoid overfitting of the analytical model. The criteria containing fold change > 1.50 or < 0.67 and *P* < 0.05 was served as the threshold value for selection of differential lipid species between two groups. MetaboAnalyst 5.0 software was used to conduct the ROC analysis of differential lipid species to explore the potential biomarkers to differentiate CA from CRC. Furthermore, the potential mechanism of CA canceration was explored *via* the KEGG and Wiki pathway database with the differential lipid species.

## Results

### Differences of serum lipid profiles between CA and CRC

**Figure 1:**
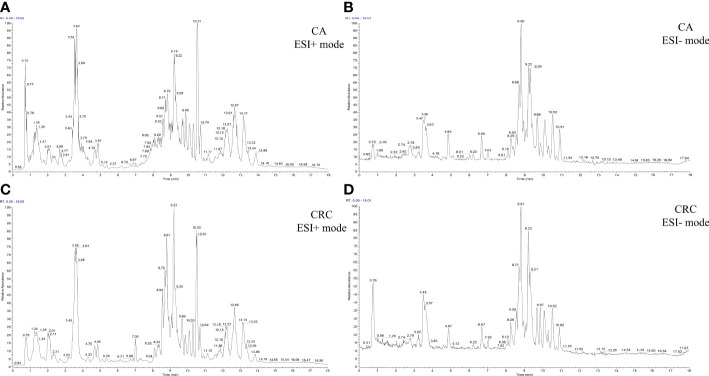
The TIC chromatography of CA and CRC groups in both ESI modes. TIC chromatography for serum lipidomics of CA in ESI+ and ESI− modes, respectively **(A, B)**; TIC chromatography for serum lipidomics of CRC in ESI+ and ESI− modes, respectively **(C, D)**.

**Figure 2:**
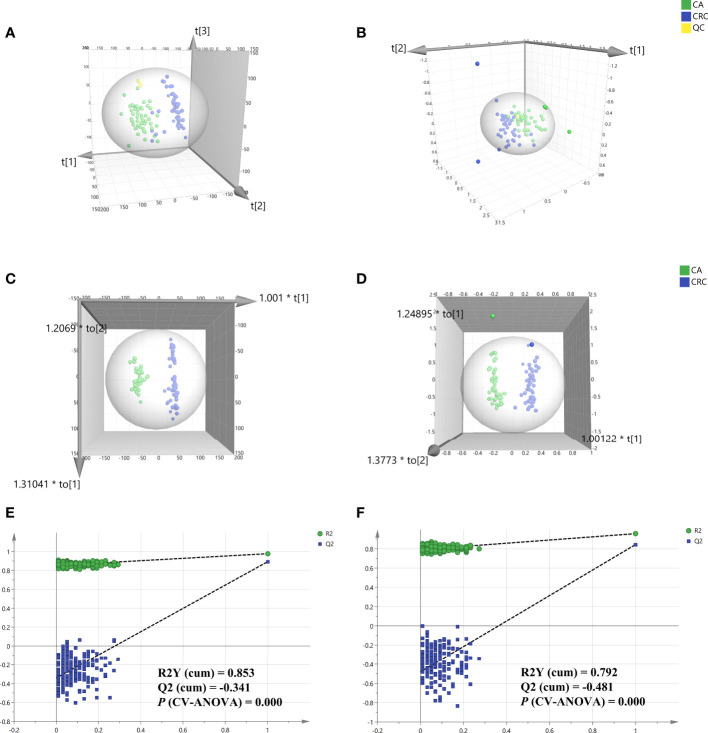
Multivariate statistical analysis of differential lipid features between CA and CRC groups in both ESI modes. PCA analysis of two groups in ESI+ and ESI- modes, respectively **(A, B)**; OPLS-DA analysis of the two groups in ESI+ and ESI− modes, respectively **(C, D)**; Overfitting test for OPLS-DA model in ESI+ and ESI− modes, respectively **(E, F)**.

Total ion chromatography (TIC) of the lipidomic study provided an overview of the general differences in the lipid profiles between the two groups. Through direct observation of serum lipid profiles, a certain difference between CA and CRC groups by TIC chromatography in both electrospray ionization (ESI) modes became evident (**Figures 1A–D**). A PCA model was established to analyze the stability of the instrument system and detection method as well as the distribution trend of samples in different groups. The three-dimensional score plots of the PCA analysis showed that QC samples were clustered closely in both ESI modes, indicating that the analysis system and detective method presented good stability and reproducibility during the batch analysis and satisfied the requirements of lipidomic analysis as well as illustrating no obvious drift of lipidomic features (**Figures 2A, B**). The samples of CA and CRC groups showed relative clustering located on two sides of the score plots, suggesting substantial differences of serum lipidomic profiles between the two groups in both ESI modes, indirectly reflecting differences in their lipid metabolism (**Figures 2A, B**). Moreover, in comparison with the ESI− mode, a more distinct trend of separation between sample clusters of CA and CRC groups was depicted in ESI+ mode (**Figures 2A, B**). Most of the samples were within the 95% confidence interval and only a few were outside the 95% confidence interval owing to a relatively discrete trend, possibly due to the existence of large individual differences (**Figures 2A, B**). In addition, the peak areas of each lipid feature in the quality control group were extracted and the RSD values for most of them were less than 20.00%, suggesting that the analysis system had good stability and reproducibility throughout the entire analytical process.

To determine the maximum differences in serum lipid profiles between CA and CRC groups, an OPLS-DA model was constructed by the all-inclusive array of lipid features based on PCA analysis to screen for potential biomarkers. Compared with PCA analysis, the three-dimensional score plots of OPLS-DA analysis illustrated that the difference of lipid profiles between the CA and CRC groups was more striking (**Figures 2C, D**). The two sample clusters were clearly separated at ESI+ mode with good parameters [R2X (cum) = 0.336, R2Y (cum) = 0.976, Q2 (cum) = 0.893] and ESI− mode [R2X (cum) = 0.535, R2Y (cum) = 0.957, Q2 (cum) = 0.844], which revealed apparent changes of lipid metabolism between the two groups (**Figures 2C, D**). This range of parameter values indicated satisfactory explanation and predictability ability for this model. In addition, the reliability and suitability of the OPLS-DA model were further analyzed and evaluated by 200 permutations test. The results provided proof that the model was rational and not overfitting for the data analysis with the values of R2 (0.853 and 0.792), Q2 (−0.341 and −0.481), and *P* (CV-ANOVA) (0.000 and 0.000) in ESI+ and ESI− model, respectively (**Figures 2E, F**).

### Screening and identification of lipid biomarkers to differentiate CA from CRC

**Figure 3:**
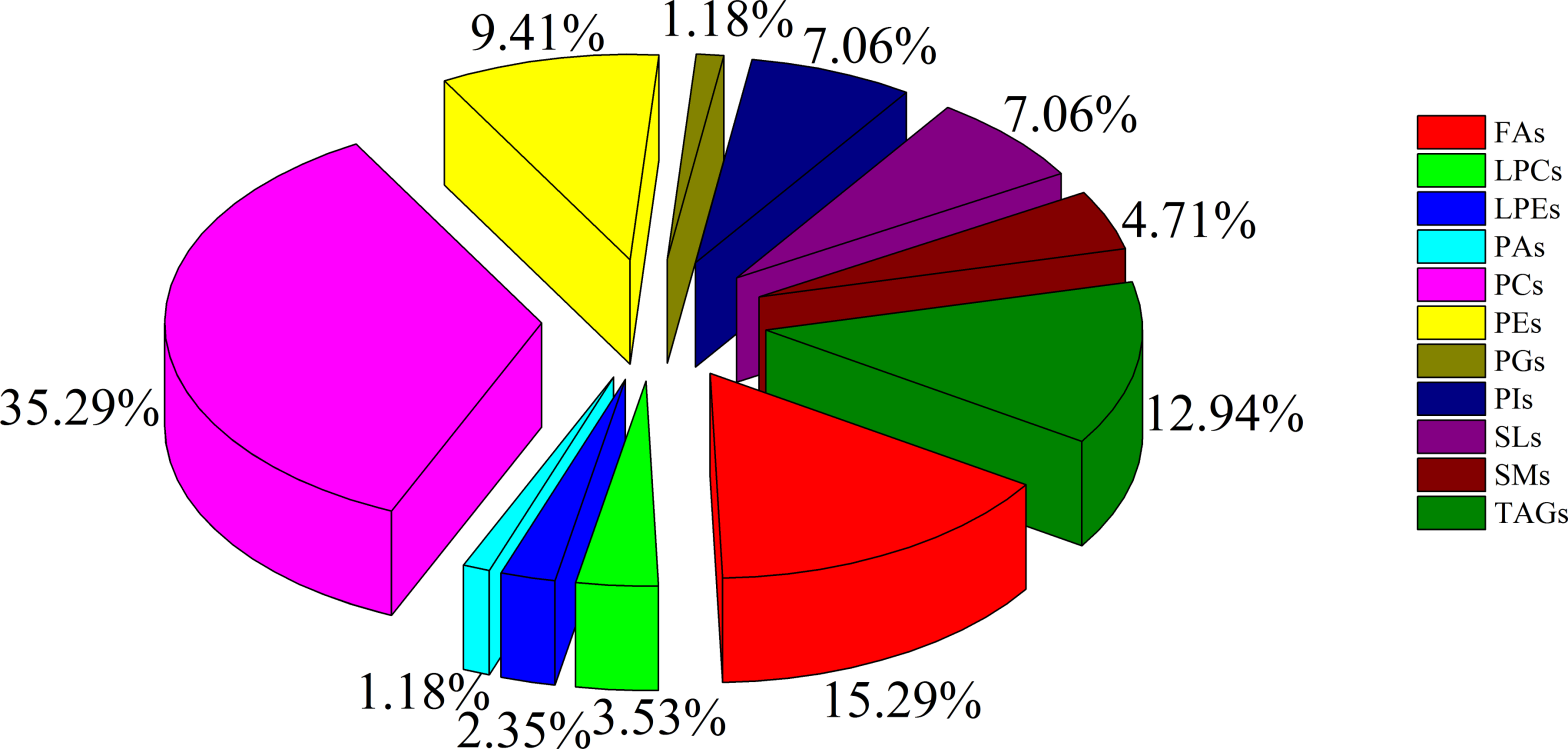
The constituent ratio of serum differential lipid species between CA and CRC groups. Abbreviations: TAG, triacylglycerol; FA, fatty acid; LPC, lysophosphatidylcholine; PG, phosphatidylglycerol; PC, phosphatidylcholine; PE, phosphatidylethanolamine; SM, sphingomyelin; LPE, lysophosphatidylethanolamine; PA, phosphatidic acid; SL, sphingolipid; PI, phosphatidylinositol.

**Figure 4:**
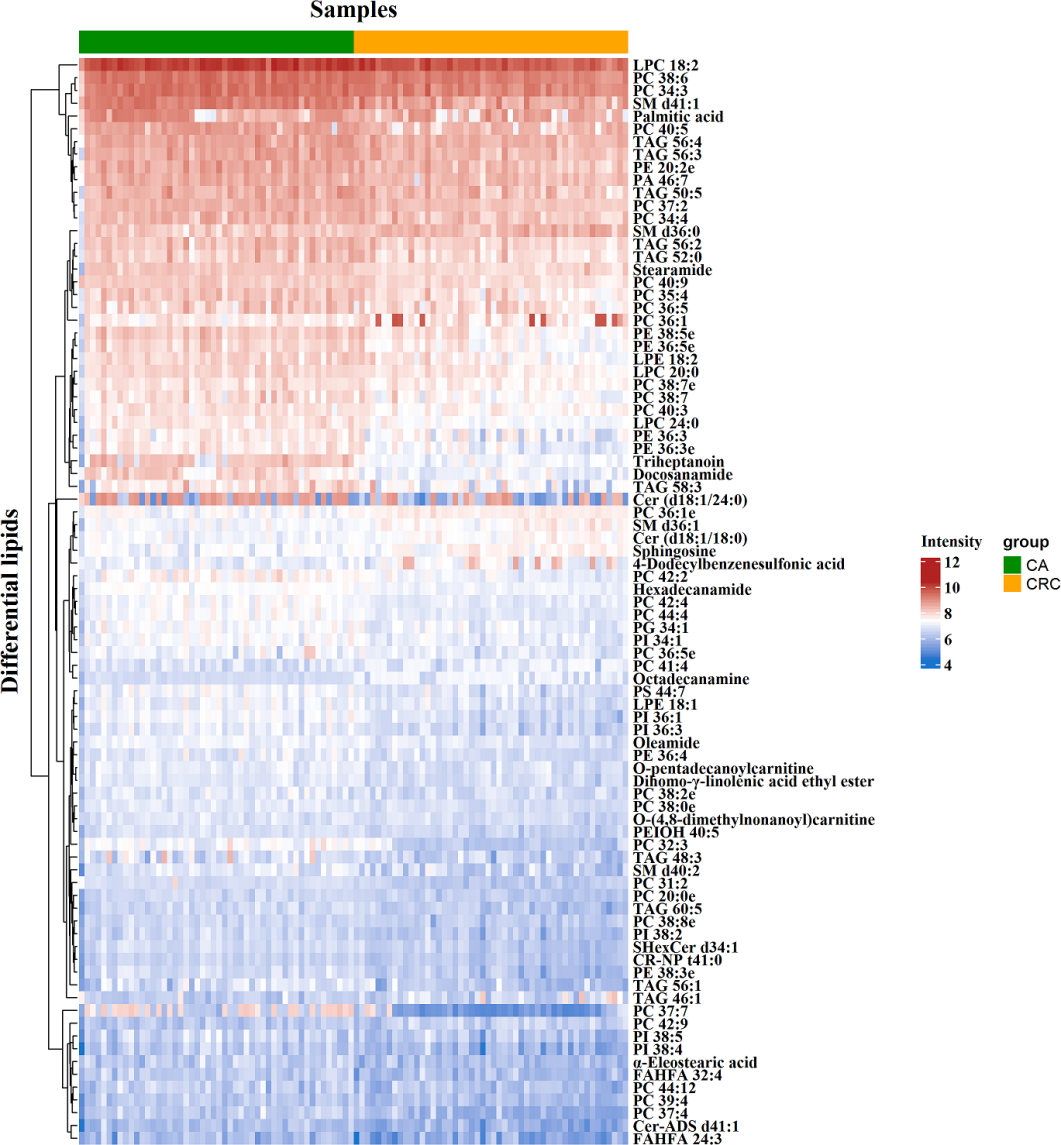
Clustering heatmap analysis of 85 differential lipid species between CA and CRC groups. The color bars represented the log10 value of the ratio for each lipid species and only statistically significant changes were shown (fold change > 1.50 or < 0.67 and *P* < 0.05).

To minimize false positives, according to the multivariate statistical analysis of serum lipid profiles between the two groups, the standard of fold change > 1.50 or < 0.67 and *P* < 0.05 was further used to screen the differential lipid species. In total, 85 differential lipid species were selected and identified (including 27 in ESI− mode and 58 in ESI+ mode) (**Table S1**). These differential lipid species included PCs: 35.29%, FAs: 15.29%, TAGs: 12.94%, PEs: 9.41%, PIs: 7.06%, SLs: 7.06%, SMs: 4.71%, LPCs: 3.53%, LPEs: 2.35%, PAs: 1.18%, and PGs: 1.18% (**Figure 3**). The percentage of PCs was the principal component with greater than 35.20%, followed by FAs and TAGs. Therefore, these three lipid types accounted for 63.52% of the total differential lipid species assessed, suggesting that dysregulation of PCs, FAs, and TAGs metabolism may be critical factors in the malignant transformation of CA to CRC. Furthermore, compared with the CA group, most of the differential lipid species were significantly downregulated in the serum of the CRC group, whereas only 11 were significantly upregulated in the CRC group (**Table S1**). To further investigate the level distribution of differential lipid species in individual samples, we performed a clustering heatmap analysis of differential lipid species between the two groups. The samples from the CA and CRC groups presented good clustering, implying that the intra-group difference and inter-group proximity of these lipid profiles were relatively pronounced (**Figure 4**). Similar to the results presented in **Table S1**, the levels of most differential lipid species dominated by PCs, FAs, and TAGs showed significant downregulation in the CRC group compared with the CA group (**Figure 4**). Taken together, PCs, FAs, and TAGs were considered the main influencing factors instigating the conversion of CA into CRC.

### Evaluation of discriminant performance of serum differential lipid species between CA and CRC

**Figure 5:**
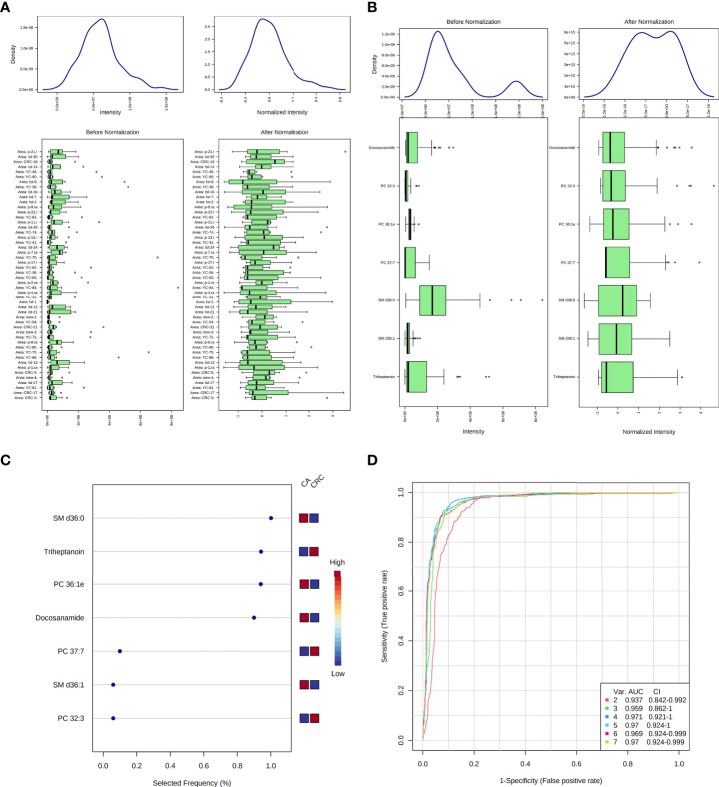
Data normalization and multivariate ROC analysis of differential lipid species between CA and CRC groups. Normalization of samples **(A)**; normalization of differential lipid species between CA and CRC groups **(B)**; the selected frequency of differential lipid species during multivariate ROC analysis by SVM algorithm **(C)**; the multivariate ROC analysis of differential lipid species with high differentiate performance for CA and CRC groups **(D)**.

**Figure 6:**
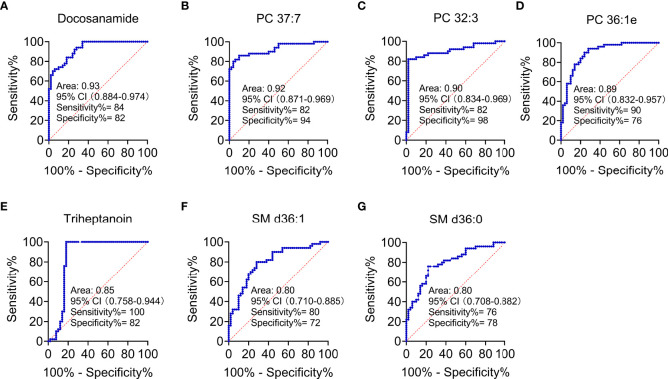
Performance evaluation of differential lipid species between CA and CRC groups. Differential lipids between CA and CRC included Docosanamide **(A)**, PC 37:7 **(B)**, PC 32:3 **(C)**, PC 36:1e **(D)**, Triheptanoin **(E)**, SM d36:1 **(F)**, and SM d36:0 **(G)** with the AUC > 0.80 in ROC analysis.

**Figure 7:**
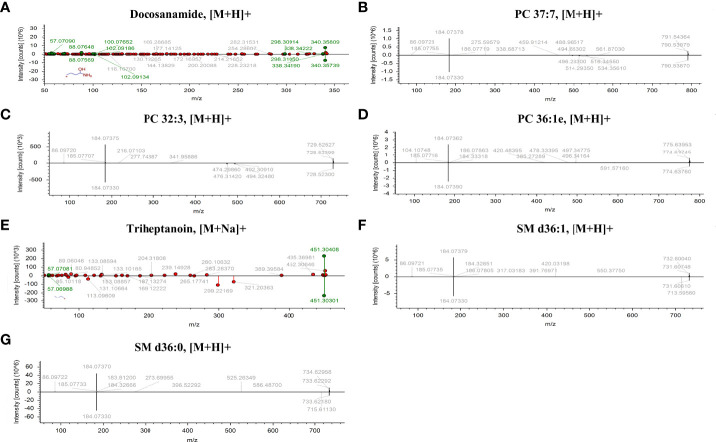
The identification of seven differential lipid species with high discriminate performance for CA and CRC. Differential lipids between CA and CRC including Docosanamide **(A)**, PC 37:7 **(B)**, PC 32:3 **(C)**, PC 36:1e **(D)**, Triheptanoin **(E)**, SM d36:1 **(F)**, and SM d36:0 **(G)** were preliminarily identified with the adduct forms of [M+H]+ and [M+Na]+.

According to MetaboAnalyst 5.0 software, biomarker analysis based on ROC results has served as a reliable tool to assess the diagnostic and discriminate performance of metabolites or proteins by maximizing the area under the ROC curve (AUC). The AUC was calculated by the trapezoidal method to select the most suitable cutoff point optimizing sensitivity and specificity of each metabolite. Prior to ROC analysis, the sum normalization and autoscaling of lipidomic data were performed to effectively decrease the influence of individual differences and systematic errors (**Figures 5A, B**). Ultimately, seven differential lipid species were identified with excellent discrimination performance (AUC > 0.80, ranging from 0.80 to 0.93), with high sensitivity (0.76 to 1.00) and specificity (0.72 to 0.98) (**Figures 6A–G**). Their identification results were achieved by matching high-resolution MS, MS/MS fragments, and RT with Thermo mzCloud and mzVault with Lipidblast database (**Figures 7A–G**). Among them, docosanamide had the highest AUC values [AUC = 0.93, 95% CI (0.884–0.974)], indicating excellent discriminative performance for CA and CRC groups, whereas SM d36:1 and SM d36:0 had relatively lower AUC values (AUC = 0.80) (**Figures 6A, F, G**). To extend the search for the potential lipid biomarkers with the highest performance in discriminating CA from CRC, a multivariate ROC curve-based analysis along with a support vector machine (SVM) algorithm was employed to perform the automated selection of the best lipid combinations. Interestingly, the seven differential lipid species were confirmed to be the most important and frequently selected variables during the panel exploration analysis, suggesting that they had high performance in distinguishing CA from CRC (**Figure 5C**). **Figure 5D** showed the ROC curves and the area under ROC curve (AUC) values of the classification models built by the top 2, 3, 4, 5, 6, and 7 of differential lipid species. From the models, the best potential biomarker panel was the combination composed of the top four differential lipid species (AUC = 0.971, 95%CI = 0.921–1.000). Therefore, a biomarker-panel of these four differential lipid species (docosanamide, PC 36:1e, triheptanoin, and SM d36:0) could act as a potential biomarker for malignant transformation of CA to develop into CRC.

### Trend of differential lipid species with high performance for the distinction between CA and CRC

**Figure 8:**
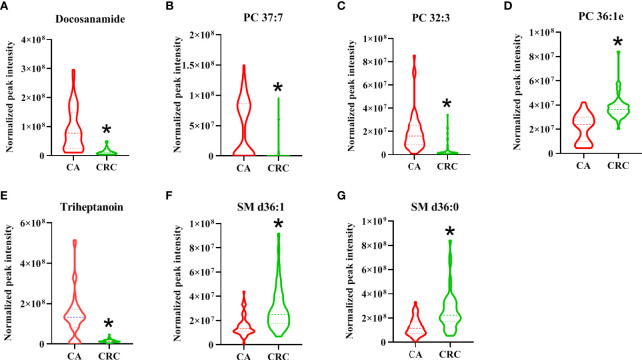
The change trend of seven differential lipid species with high performance for the discrimination of CA and CRC groups. The levels of differential lipid species between CA and CRC groups were displayed with mean ± SEM. The “★” represented statistical significance of the variate with *P* < 0.05 between two groups. Among them, the levels of differential lipids of Docosanamide **(A)**, PC 37:7 **(B)**, PC 32:3 **(C)**, and Triheptanoin **(E)** showed significant decrease, whereas those of PC 36:1e **(D)**, SM d36:1 **(F)**, and SM d36:0 **(G)** exhibited obvious increase in the patients with CRC compared to patients with CA.

As shown in the lipid profiles analysis, there were significant differences between the CA and CRC groups, which included 85 differential lipid species (30 PCs, 13 FAs, 11 TAGs, eight PEs, six PIs, six SLs, four SMs, three LPCs, two LPEs, one PA, and one PG; **Table S1**). From the above ROC analysis, the change trend for the seven potential lipid biomarkers (three PCs, two FAs, and SMs), with good discriminative performance between the two groups, was further explored. The results revealed that four lipid species—docosanamide, PC 37:7, PC 32:3, and triheptanoin—were significantly upregulated in the CA group, whereas the other three lipid species—PC 36:1e, SM d36:1, and SM d36:0—were remarkably downregulated in the CA group compared with the CRC group (**Figures 8A–G**). Among them, docosanamide, PC 37:7, and triheptanoin exhibited the most significant change trends with fold change > 5 (**Table S1**), consistent with the clustering heatmap of differential lipid species between groups (**Figure 4**). These differential lipid species were mainly involved in the metabolic pathways of FA, PC, and TAG metabolism. Taken together, FAs and PCs were the main dysregulated lipid biomarkers to distinguish between CA and CRC groups. The perturbation of PC, FA, and TAG metabolism was closely associated with CA cancerization.

## Discussion

To our knowledge, the current study represents the first prospective analysis of lipidomics for patients with CA and CRC. It presents a comprehensive insight into the lipidomics associated with CA and CRC by using UHPLC-HRMS, providing both a highly powered dataset and reliable analysis for results. In the present study, there were 85 differential lipid species identified between the two groups, seven of which were determined to show good discriminate efficacy (AUC > 0.80). Furthermore, the biomarker-panel of four lipid species demonstrated the highest selected frequency and AUC value to serve as a potential biomarker to differentiate CA from CRC. In addition, among the 85 differential lipid species, PCs, FAs, and TAGs were the main components with a total constituent ratio of more than 63.50%. Concluding from these results, dysregulation of metabolic homeostasis of PCs, FAs, and TAGs could be involved in the malignant transformation of CA into CRC. These findings should contribute to the practical knowledge directed toward identification of the most relevant biomarkers in CA and CRC. These data were largely in agreement with previous findings that reflected the key role of lipid-mediated metabolic pathways in CRC early diagnosis, remedy, and prognosis.

Lipid species are an integral part of cells, which play pivotal roles throughout the cellular life cycle due to their wide range of functions, such as in cell survival, proliferation, signal transduction, and apoptosis (24). Lipid metabolism is a complex process involving lipid uptake, transport, synthesis, and degradation (25). Accumulating studies have shown that dysregulated lipid metabolism causes abnormalities of membrane composition, protein distribution and functions, gene expression, and cellular functions and further leads to the occurrence and development of many cancers, such as breast, prostate, ovarian, and colorectal (24–26). Relevant to the present study, dyslipidemia has also been recognized as an important risk factor of CA and CRC (26, 27).

It is well known that PCs are the most abundant glycerophospholipids in eukaryotic membranes, abnormalities of which are related to tumor cell proliferation and signal transduction (24, 28). Metabolic disorders of PCs have been found in various tumors, and some PCs have reportedly shown significant potential as tumor biomarkers (24, 26, 29). In recent studies, the expression of PCs in cancer patients was shown to be decreased (30–32), and this downregulation might be due to overexpression of phospholipase A2 (PLA2) in tumors (33), which causes hydrolysis of most PCs into FAs and LPCs. In addition, disorders in synthesis might also be responsible for the decline of serum PC content (34). In plasma metabolomics, 48 differential metabolites, principally LPCs and PCs, were identified between CA and CRC, and their levels were previously shown to be downregulated in CRC (35). In our previous study, PCs were dominant components of differential lipid species between CA and normal controls (NR) and were significantly reduced in CA; this implicated abnormal PC metabolism as a likely contributor to the formation of CA (26). Similarly, the present study found that PCs were notably downregulated in patients with CRC compared with CA patients (**Table S1**, **Figures 3**, **4**). It might be inferred, therefore, that levels of PCs are inversely proportional to the severity of CRC and can potentially act as the main putative biomarkers for discriminating CA from CRC. Among all differential PCs, we also found that PC 37:7 was sharply decreased in CRC (fold change > 5) and had a high discriminative ability (AUC = 0.92), which may be a potential biomarker for CA carcinogenesis (**Table S1**, **Figures 6**, **8B**).

Apart from PCs, FAs are also potential diagnostic markers for distinguishing CRC or CA from normal, according to previous reports (36, 37). Many lipid species are usually synthesized from FAs, which are a diverse class of molecules consisting of hydrocarbon chains of varying lengths and degrees of desaturation. FAs form the hydrophobic tails of phospholipids and glycolipids, which, along with cholesterol, are the major components of biological membranes. Dysregulation of FA synthesis and metabolism can lead to cancer through many pathways, not only as the building blocks for membrane synthesis or substrates for ATP synthesis during cell growth but also participating in the regulation of signaling pathways involved in cell proliferation and survival (38). Beyond tumorigenesis, lipid controlled signaling processes also play an important role in cancer progression and metastasis, which are the key steps advancing toward cancer-related death. Multiple prior reports have indicated that cancer cells express higher levels of FAs than corresponding normal cells, fulfilling the increased requirement of lipid species that cancer cells need to meet the energy demands for upregulated synthesis, signal transduction, and cell membrane formation (26, 39, 40). However, compared with CA, although octadecanamine and 4-dodecylbenzenesulfonic acid were significantly increased in CRC, most FAs showed distinct downregulation in CRC (**Table S1**). A possible explanation is that cancer cells within tissues uptake a large number of FAs, resulting in correspondingly decreased blood levels of FA. In particular, our previous study showed that docosanamide and triheptanoin were found to be potential biomarkers for the diagnosis of CAA (12) and CA (26), respectively, and their AUC values were more than 0.85. Similarly, in this research, we also found that docosanamide and triheptanoin presented significant differences between CA and CRC. Both of them have good discriminative performance through ROC analysis, especially docosanamide has the highest AUC, reaching 0.93 (**Table S1**, **Figures 6A, E**); hence, docosanamide and triheptanoin could serve as the putative biomarkers for the colorectal “adenoma to carcinoma” sequence.

FAs are also stored in adipose tissue as TAGs, and when energy is depleted, the TAGs are degraded to release FAs (25). Hence, the metabolic perturbation of TAG generally dysregulates FA metabolism. Although some studies have reported a positive association between circulating TAGs and the risk of CA and CRC, these findings have been inconsistent (41). As a class of glycerides, disturbances in serum or plasma TAG levels have a close relationship with an increased risk of CA and CRC progression (42–44). In our recent study, TAGs were the predominant components of differential lipid species between CA and NR, indicating that their abnormal metabolism should contribute to the formation of CA (26). In addition, as the severity of CA progressed, it may transform into a colorectal advanced adenoma (CAA) and, as such, would also be considered an ideal target for early prevention of CRC. In the previous investigation, we found that TAGs were the major dysregulated lipid species of CAA (12). In the present study, we also found that TAGs were the main dysregulated lipid species in CA progression to CRC (**Table S1**, **Figures 3**, **4**), lending further evidence that the dyshomeostasis of TAG metabolism was involved in CRC progression.

In addition, studies have demonstrated that SMs can regulate critical aspects of cell division, proliferation, and chemotaxis, leading to the occurrence and progression of cancer (45). Moro et al. disclosed that the level of SMs in breast cancer were significantly higher than that in peritumor tissue and normal tissue by LC-MS/MS (46). Alkaline sphingomyelinase converts SMs into a molecule with ceramide that promotes apoptosis (47). Mika et al. found that the activity of alkaline sphingomyelinase in CRC tissues was significantly reduced, which could increase SM content and decrease ceramide levels, thereby preventing apoptosis and promoting the development of CRC (48). Similarly, in our study, SMs also can be employed as the main dysregulated biomarkers to help distinguish CA from CRC. In particular, the SM d36:1 and SM d36:0 presented satisfactory discriminative performance (AUC = 0.80) and the same change trend as the above studies (**Table S1**, **Figures 6**, **8F, G**). In addition, other studies have identically proposed the SM as potential lipid biomarker for diagnosis of CRC. Shen et al. performed a 2D LC-QToF/MS-based lipidomic analysis indicated that SM 42:2 and SM 38:8 showed excellent ability (AUC > 0.90) to differentiate patients with CRC from healthy controls (22). Likewise, SM d18:1/16:0 was found to be metabolically dysregulated in exosomes derived from colon cancer cell (49) and could act as a biomarker for the diagnosis of CRC by targeted lipidomics (50).

Therefore, compiling all of the presented evidence, our results indicated that the dysregulated lipid metabolism of PCs, FAs, and TAGs should be closely connected with the malignant transformation process from CA to CRC, and that the biomarker-panel of four lipid species (docosanamide, PC 36:1e, triheptanoin, and SM d36:0) could serve as candidate biomarkers for differentiating CA from CRC. Nevertheless, the discriminative ability of lipid biomarkers for CA and CRC still needs external verification, probably through a stricter design and larger sample sizes.

## Conclusions

To our knowledge, this was the first study to explore lipid biomarkers to distinguish between CA and CRC based on serum lipidomics by the UHPLC-HRMS technique. The serum lipid profiles displayed a significant difference between CA and CRC, mainly due to 85 differential lipid species. Among them, PCs, FAs, and TAGs were the major components, suggesting that the abnormal metabolism of these lipids could be involved in the malignant transformation of CA. In addition, seven differential lipid species showed good discriminative efficacy for CA and CRC as potential biomarkers. Moreover, four of them (docosanamide, SM d36:0, PC 36:1e, and triheptanoin) were selected as a potential biomarker-panel with outstanding performance in distinguishing CA from CRC, based on the SVM classification model. The overarching theme of the present study was that the mechanism of malignant transformation of CA to CRC is associated with abnormal lipid metabolism. From a clinical perspective, the discovery of putative lipid biomarkers that could reliably discriminate CA from CRC might hold promise as a novel early screening test for CRC.

## Data availability statement

The original contributions presented in the study are included in the article/**supplementary material**. Further inquiries can be directed to the corresponding author.

## Ethics statement

The studies involving human participants were reviewed and approved by The Ethics Committee of the People’s Hospital of Guangxi Zhuang Autonomous Region. The patients/participants provided their written informed consent to participate in this study.

## Author contributions

Conceptualization and investigation, HC and JZ. Writing—original draft preparation, HC. Data curation, JZ and HZ. Methodology and formal analysis, HZ and YL. Visualization, YZ and PZ. Writing—review and editing, project administration, and funding acquisition, QZ. All authors have reviewed and agreed to the published version of this manuscript. All authors contributed to the article and approved the submitted version.

## Funding

This research was supported financially by the Fund Project of Guangxi University (No. A3370051006), the Bama County Program for talents in science and technology (No. AE33700024), and the Innovation Project of Guangxi Graduate Education (No. YCSW2022076).

## Acknowledgments

The authors sincerely wish to thank the support and help of workers in State Key Laboratory for Conservation and Utilization of Subtropical Agro-Bioresources of Guangxi University. The authors would like to express their gratitude to EditSprings (https://www.editsprings.cn) for the expert linguistic services provided.

## Conflict of interest

The authors declare that the research was conducted in the absence of any commercial or financial relationships that could be construed as a potential conflict of interest.

## Publisher’s note

All claims expressed in this article are solely those of the authors and do not necessarily represent those of their affiliated organizations, or those of the publisher, the editors and the reviewers. Any product that may be evaluated in this article, or claim that may be made by its manufacturer, is not guaranteed or endorsed by the publisher.

## Abbreviations

UHPLC, ultrahigh-performance liquid chromatography; HPLC, high-performance liquid chromatography; PCA, principal component analysis; OPLS-DA, orthogonal partial least squares discriminant analysis; SVM, support vector machine; TIC, total ion chromatography; ROC, receiver operating characteristic; QC, quality control; CI, confidence interval; ESI, electrospray ionization; HRMS, high-resolution mass spectrometry; CA, colorectal adenoma; CAA, colorectal advanced adenoma; NR, normal control; CRC: colorectal cancer; AUC, area under the curve; RT, retention time; *m/z*, mass to charge ratio; S/N, signal-noise ratio; RSD, relative standard deviation; TAGs, triacylglycerols; LPCs, lysophosphatidylcholines; PEs, phosphatidylethanolamines; PAs, phosphatidic acids; PGs, phosphatidylglycerols; PIs, phosphatidylinositols; SLs, sphingolipids; PCs, phosphatidylcholines; SMs, sphingomyelins; FAs, fatty acids; LPEs, lysophosphatidylethanolamines.
