# Supplementary material for: UHPLC-HRMS–based serum lipisdomics reveals novel biomarkers to assist in the discrimination between colorectal adenoma and cancer
Source: Front Oncol. 2022 Jul 28;12:934145. doi: 10.3389/fonc.2022.934145 (PMC9366052; doi:10.3389/fonc.2022.934145)
Supplement: Supplementary file 1 [file Table_1.docx]

Supplementary Material

**Table S1.** Differential lipid species (N = 85) between CA and CRC groups in both ESI modes.

| **Lipid species** | **Formula** | **Scan mode** | **Detective *m/z*** | **RT (min)** | ***P* value** | **Fold change** |
| --- | --- | --- | --- | --- | --- | --- |
| α-Eleostearic acid | C18 H30 O2 | ESI+ | 279.23227 | 6.49 | 0.000 | 0.43 |
| Triheptanoin | C24 H44 O6 |  | 451.30408 | 5.32 | 0.000 | 0.11 |
| TAG 56:2 | C59 H110 O6 |  | 932.86536 | 14.57 | 0.000 | 0.53 |
| TAG 60:5 | C63 H112 O6 |  | 982.88098 | 14.54 | 0.000 | 0.51 |
| TAG 58:3 | C61 H112 O6 |  | 958.88074 | 14.77 | 0.009 | 0.45 |
| TAG 56:4 | C59 H106 O6 |  | 928.83374 | 13.27 | 0.000 | 0.62 |
| TAG 56:3 | C59 H108 O6 |  | 930.84888 | 13.99 | 0.000 | 0.58 |
| TAG 56:1 | C59 H112 O6 |  | 934.87665 | 14.70 | 0.001 | 0.44 |
| TAG 52:0 | C55 H106 O6 |  | 830.83447 | 14.67 | 0.019 | 0.59 |
| TAG 48:3 | C51 H92 O6 |  | 818.72369 | 11.59 | 0.014 | 0.16 |
| TAG 46:1 | C49 H92 O6 |  | 794.72467 | 12.22 | 0.032 | 3.72 |
| Stearamide | C18 H37 N O |  | 284.29538 | 4.98 | 0.000 | 0.57 |
| Sphingosine | C18 H37 N O2 |  | 300.32635 | 2.79 | 0.000 | 1.74 |
| SM d36:0 | C41 H85 N2 O6 P |  | 733.62292 | 9.40 | 0.000 | 2.01 |
| SM d41:1 | C46 H93 N2 O6 P |  | 801.68469 | 10.27 | 0.000 | 0.35 |
| PC 36:1e | C44 H88 N O7 P |  | 774.63745 | 9.70 | 0.000 | 1.79 |
| PC 40:3 | C48 H90 N O8 P |  | 840.64874 | 9.86 | 0.000 | 0.58 |
| PC 42:2 | C50 H96 N O8 P |  | 870.69550 | 10.50 | 0.000 | 0.41 |
| PC 41:4 | C49 H90 N O8 P |  | 852.68213 | 10.14 | 0.000 | 1.64 |
| PC 40:9 | C48 H78 N O8 P |  | 828.55286 | 8.30 | 0.000 | 0.62 |
| PC 40:5 | C48 H86 N O8 P |  | 836.60870 | 9.08 | 0.000 | 0.45 |
| PC 38:7e | C46 H80 N O7 P |  | 790.57538 | 8.59 | 0.001 | 0.65 |
| PC 38:2e | C46 H90 N O7 P |  | 800.65918 | 10.12 | 0.000 | 0.55 |
| PC 37:4 | C45 H82 N O8 P |  | 796.57971 | 8.30 | 0.003 | 0.36 |
| PC 36:5e | C44 H80 N O7 P |  | 766.57550 | 8.72 | 0.008 | 0.48 |
| PC 44:12 | C52 H80 N O8 P |  | 878.57056 | 7.85 | 0.001 | 0.50 |
| Palmitic acid | C16 H32 O2 |  | 274.27435 | 1.19 | 0.001 | 0.53 |
| O-pentadecanoylcarnitine | C22 H43 N O4 |  | 386.32773 | 1.53 | 0.000 | 0.62 |
| Oleamide | C18 H35 N O |  | 282.27966 | 10.68 | 0.000 | 0.65 |
| Octadecanamine | C18 H39 N |  | 270.31567 | 3.25 | 0.000 | 2.74 |
| O-(4,8-dimethylnonanoyl) carnitine | C18 H35 N O4 |  | 330.26450 | 1.04 | 0.000 | 0.65 |
| SM d36:1 | C41 H83 N2 O6 P |  | 731.60748 | 8.95 | 0.000 | 1.89 |
| SM d40:2 | C45 H89 N2 O6 P |  | 785.65851 | 11.28 | 0.001 | 0.52 |
| Hexadecanamide | C16 H33 N O |  | 256.26404 | 2.62 | 0.000 | 0.64 |
| Docosanamide | C22 H45 N O |  | 340.35809 | 6.91 | 0.000 | 0.15 |
| Dihomo-γ-linolenic acid ethyl ester | C22 H38 O2 |  | 335.29327 | 2.41 | 0.000 | 0.64 |
| Cer (d18:1/24:0) | C42 H83 N O3 |  | 650.64496 | 10.84 | 0.000 | 0.44 |
| Cer (d18:1/18:0) | C36 H71 N O3 |  | 566.55072 | 9.73 | 0.000 | 1.68 |
| PC 34:4 | C42 H76 N O8 P |  | 754.53900 | 8.12 | 0.001 | 0.63 |
| LPC 24:0 | C32 H66 N O7 P |  | 608.46625 | 7.84 | 0.000 | 0.39 |
| PC 31:2 | C39 H74 N O8 P |  | 716.56000 | 8.60 | 0.006 | 0.34 |
| PC 34:3 | C42 H78 N O8 P |  | 756.55426 | 8.29 | 0.000 | 0.59 |
| PC 36:1 | C44 H86 N O8 P |  | 788.61700 | 9.56 | 0.002 | 23.35 |
| LPE 18:2 | C23 H44 N O7 P |  | 478.29074 | 2.91 | 0.000 | 0.50 |
| LPC 18:2 | C26 H50 N O7 P |  | 520.34052 | 2.76 | 0.000 | 0.47 |
| LPC 20:0 | C28 H58 N O7 P |  | 552.40338 | 6.06 | 0.000 | 0.54 |
| PC 20:0e | C28 H58 N O7 P |  | 552.40582 | 5.83 | 0.000 | 0.57 |
| PC 38:0e | C46 H94 N O7 P |  | 804.68542 | 10.66 | 0.000 | 0.63 |
| PE 36:4 | C41 H74 N O8 P |  | 740.52350 | 8.75 | 0.002 | 0.52 |
| PC 38:6 | C46 H80 N O8 P |  | 806.57025 | 8.30 | 0.000 | 0.63 |
| PC 42:4 | C50 H92 N O8 P |  | 866.66479 | 10.08 | 0.000 | 0.47 |
| TAG 50:5 | C53 H92 O6 |  | 825.69653 | 11.46 | 0.008 | 0.62 |
| PC 37:7 | C45 H76 N O8 P |  | 790.53979 | 7.81 | 0.000 | 0.10 |
| PC 44:4 | C52 H96 N O8 P |  | 894.69641 | 10.45 | 0.000 | 0.42 |
| PC 38:7 | C46 H78 N O8 P |  | 804.55463 | 7.89 | 0.000 | 0.40 |
| PC 42:9 | C50 H82 N O8 P |  | 856.64368 | 9.53 | 0.004 | 1.54 |
| PC 37:2 | C45 H86 N O8 P |  | 800.61780 | 9.53 | 0.000 | 0.61 |
| PC 32:3 | C40 H74 N O8 P |  | 728.52399 | 8.31 | 0.000 | 0.21 |
| PS 44:7 | C50 H84 N O10 P | ESI- | 888.57019 | 9.04 | 0.000 | 0.45 |
| PI 38:2 | C47 H87 O13 P |  | 888.57019 | 9.04 | 0.000 | 0.45 |
| PI 36:1 | C45 H85 O13 P |  | 863.56616 | 9.29 | 0.000 | 0.35 |
| PC 38:8e | C46 H78 N O7 P |  | 846.56372 | 8.99 | 0.000 | 0.66 |
| PC 36:5 | C44 H78 N O8 P |  | 824.54669 | 8.43 | 0.005 | 0.57 |
| PA 46:7 | C49 H83 O8 P |  | 829.58014 | 8.94 | 0.000 | 0.65 |
| PC 39:4 | C48 H88 N O10 P |  | 868.60950 | 9.50 | 0.005 | 0.62 |
| SHexCer d34:1 | C40 H77 N O11 S |  | 778.51599 | 8.20 | 0.000 | 0.49 |
| FAHFA 32:4 | C32 H54 O4 |  | 501.39481 | 6.79 | 0.000 | 0.49 |
| FAHFA 24:3 | C24 H40 O4 |  | 391.28580 | 4.73 | 0.000 | 0.41 |
| Cer-ADS d41:1 | C41 H81 N O4 |  | 650.61047 | 10.49 | 0.001 | 0.46 |
| 4-Dodecylbenzenesulfonic acid | C18 H30 O3 S |  | 325.18448 | 2.64 | 0.001 | 6.12 |
| PE 20:2e | C25 H48 N O7 P |  | 504.30997 | 2.78 | 0.000 | 0.49 |
| PC 35:4 | C43 H78 N O8 P |  | 831.60521 | 8.44 | 0.000 | 0.45 |
| LPE 18:1 | C23 H46 N O7 P |  | 478.29391 | 3.91 | 0.000 | 0.52 |
| PG 34:1 | C40 H77 O10 P |  | 747.51691 | 9.00 | 0.001 | 0.64 |
| PE 38:5e | C43 H78 N O7 P |  | 750.54559 | 9.62 | 0.000 | 0.38 |
| PE 36:5e | C41 H74 N O7 P |  | 722.51422 | 9.15 | 0.000 | 0.38 |
| CR-NP t41:0 | C42 H85 N O6 |  | 698.63416 | 10.55 | 0.000 | 0.58 |
| PE 36:3 | C41 H76 N O8 P |  | 740.52627 | 9.00 | 0.000 | 0.58 |
| PE 36:3e | C41 H78 N O7 P |  | 726.54504 | 9.71 | 0.000 | 0.38 |
| PE 38:3e | C43 H82 N O7 P |  | 754.57587 | 10.13 | 0.000 | 0.38 |
| PI 38:5 | C47 H81 O13 P |  | 883.53564 | 8.53 | 0.001 | 0.47 |
| PI 36:3 | C45 H81 O13 P |  | 859.53516 | 8.42 | 0.000 | 0.38 |
| PI 34:1 | C43 H81 O13 P |  | 835.53510 | 8.80 | 0.000 | 0.56 |
| PI 38:4 | C47 H83 O13 P |  | 885.54956 | 8.56 | 0.000 | 0.39 |
| PEIOH 40:5 | C45 H79 O8 P |  | 776.54083 | 9.09 | 0.000 | 0.62 |

Fold change: the arithmetic means of peak intensity of CRC/CA.

Abbreviations: RT, Retention time; *m/z*, mass charge ratio; TAG, Triacylglycerol; Cer, Ceramide; FA, Fatty acid; SM, Sphingomyelin; LPC, Lysophosphatidylcholine; PG, Phosphatidylglycerol; PA, Phosphatidic Acid; PC, Phosphatidylcholine; PE, Phosphatidylethanolamine; LPE, Lysophosphatidylethanolamine; PS, Phosphatidylserine; PI, Phosphatidylinositol.
